# Thyroidectomy With or Without Nerve Identification: A Personal Experience and Technique

**DOI:** 10.7759/cureus.40312

**Published:** 2023-06-12

**Authors:** Loay S Ahmed, Fakhraddin Naser, Emad Mohammed

**Affiliations:** 1 Department of General Surgery, Kirkuk College of Medicine, Kirkuk, IRQ; 2 Department of Surgery, Azadi Teaching Hospital, Kirkuk, IRQ

**Keywords:** thyroid nerve visualization, thyroidectomy, thyroid surgery, eln injury, rln injury

## Abstract

Introduction: Careful and precise dissection of the gland, away from the typical trajectory of the recurrent and external laryngeal nerves, poses a minimal or similar risk of nerve injuries compared to directly visualizing and identifying the nerves.

Materials and methods: In a randomized controlled study involving 150 patients with various thyroid disorders who underwent different surgical procedures (total, near total, and hemi thyroidectomy), the patients were randomly assigned into two groups using a coin toss. The first group (G1) consisted of 75 patients who underwent thyroidectomy with nerve visual identification, while the second group (G2) comprised 75 patients without the requirement of nerve visualization. The aim was to determine the method with a lower risk of complications.

Results: The incidence of external laryngeal nerve palsy (ELNP) was found to be higher in G1 patients compared to G2 patients (5.3% vs 2%), while no cases of permanent recurrent laryngeal nerve (RLN) palsy were observed in either group. The frequency of total nerve injury was higher in G1, with 14 patients (10.2%), compared to G2, with eight patients (5.3%). However, there was no significant association between nerve identification and the rate of nerve injury (P value = 0.452). Among the different surgical procedures, total thyroidectomy for toxic goiter was the most common operation associated with transient external laryngeal nerve (TELN) injury and permanent external laryngeal nerve (PELN) injury.

Conclusion:* *By employing meticulous dissection techniques in proximity to the thyroid capsule, experienced surgeons can effectively reduce the risk of nerve injury, even in the absence of direct nerve visualization.

## Introduction

A thorough understanding of nerve anatomy serves as a safe guide to performing surgery with the least amount of morbidity. In contrast to the left recurrent laryngeal nerve (RLN), the right recurrent laryngeal nerve often passes behind the artery before climbing into the neck, its course being more oblique. In 25% of people, a nerve branch may cross a berry ligament, making this intersection particularly prone to damage. The vagus nerve also gives rise to the superior laryngeal nerves. Because the nerve crosses below the thyroid superior pole, the type 2a mutation, which can affect up to 20% of people, increases the risk of nerve injury [1].

Intraoperative nerve monitoring (IONM) errors occur in 3.8-23.0% [2]. IONM and visualization did not change the incidence of RLN palsy during thyroidectomy [3]. IONM is recommended for those patients with large thyroid masses, retrosternal goiters, hyperthyroidism, malignant thyroid tumors, second thyroid surgeries, non-recurrent RLN, and unilateral vocal cord paralysis [4,5]. According to several studies, postoperative RLN damage can be temporary or permanent, depending on the stringency of postoperative otolaryngologic controls. Transient injuries occur in 1% to 30% of patients, while permanent injuries occur in 0.5% to 5% [6,7]. In the largest multi-institutional prospective trial, persistent paralysis did not benefit from IONM over visual RLN identification [8]. 

IONM over visual nerve identification alone does not presently have any conclusive evidence to support its superiority or inferiority on any of the outcomes studied [9]. Hence the aim of our study is to show if there is any difference regarding the outcome of surgery whether nerves are visualized or not during the surgery.

## Materials and methods

The clinical study was done among 150 patients complaining of different thyroid disorders who underwent many types of surgical procedures (hemi and total thyroidectomy), undertaken by experienced surgeons at the surgical department of the teaching hospital of Kirkuk College of Medicine and private hospitals over the period between 2018 - Jan 2020.

We randomly divided patients by tossing the coin into two groups: In the first group (G1) of 75 patients, nerve visual identification was done using blunt dissection with a peanut swab or dissector to identify the recurrent laryngeal nerve/external laryngeal nerve (RLN/ELN). In the second group (G2) of 75 patients, we tried to be very close to the gland capsule with meticulous, delicate bloodless dissection by dividing the small ending vessels over the gland without dividing the main feeding vessels away from the gland. Regarding the ELN we ligated visible superior thyroid vessels over the lying superior pole of the gland proper without ligating the pedicle higher up away from the gland proper with no need to identify the nerves.

All patients underwent a comprehensive investigation and preparations, ensuring they were in a euthyroid state, and medical consent was obtained regarding potential post-thyroidectomy complications. Both procedures were performed by an experienced surgeon. The operative procedure began with routine positioning of the thyroid and a collar incision. Identification of both or one lobe was achieved after ligating the middle thyroid vein. Regarding the G2, the important issue is to dissect the field under a completely bloodless field and avoid cauterization at the dangerous risky zone of the nerve course, unipolar cauterization or harmonic device was used if needed till we reach the trachea. All patients were examined postoperatively for any vocal cord dysfunction at the time of recovery from anesthesia after extubation by the anesthetists and any voice tone changes in early post-operative days by an Ear Nose Throat (ENT) specialist. Exclusion criteria were patients with recurrent thyroid surgery, previous nerve injury, big goiters, and retrosternal goiter.

We utilize the chi-square and P values to determine whether there is a significant correlation between nerve injury in relation to procedure. If a result was less than 0.05, it was considered statistically significant. To determine whether there is a significant association between the frequency of nerve injury and the kind of operation, we use the Pearson correlation test.

## Results

Table 1 illustrates that the majority of patients were female in both groups (64/75; 86.6%).

**Table 1:** Comparative data between G1 and G2 patients TRL: Transient Recurrent Laryngeal Nerve PRL: Permanent Recurrent Laryngeal Nerve TEL: Transient External Laryngeal Nerve PEL: Permanent External Laryngeal Nerve

Data	G1	G2
Number of patients	75	75
Mean age	39 years	39.5 years
Male	11	11
Female	64	64
Total thyroidectomy	65 (86.6%)	40 (53.3%)
Hemi thyroidectomy	6 (8%)	7 (9.3%)
lobectomy	4 (5.3%)	28 (37.3%)
Toxic goiter	4 (5.3%)	15 (25.3%)
Nontoxic goiter	62 (82.7%)	55 (68%)
Cancer	9 (12%)	5 (6.7%)
TRL palsy	5 (6.6%)	3 (4%)
PRL palsy	0	0
TEL palsy	8 (10.6%)	3 (4%)
PEL palsy	1 (1.3%)	2 (2.6%)

The mean age was 39 years in G1 and 39.5 years in G2. Sixty-two (82.7%) patients were nontoxic in G1 and 55 (68%) patients in G2. While toxic patients formed about 4/15 (5.3%, 25%) patients in G1 andG2 respectively, cancer patients formed 9/5 (12%,6.7%) patients in G1 and G2 respectively. The majority of patients underwent total thyroidectomy in G1 (61; 81.3%), versus 40 (53.3%) patients in G2. Temporary RLNP was 6.6% vs. 4% in G1 and G2 respectively, and there was no incidence of permanent RLN palsy in either group.

Temporary ELNP in G1 patients was higher than in G2 patients (5.3% vs. 2%) while permanent palsy was higher in G2 (1.3% versus 0.6%). The frequency of total nerve injuries was higher in G1, with 14 patients (10.2%), compared to G2, with eight patients (5.3%) (Table 2). Total nerve injury was more with total thyroidectomy for toxic goiters than other types of goiter surgery in G1 (nine patients; 11.9%) (Table 3). In G2 patients, total nerve injury was more common in total thyroidectomy for toxic goiters compared to other types of goiter surgery (Table 4). We accept the null hypothesis that there is no significant correlation between the identification of the nerve and the rate of nerve injury (Table 5), but there was a strong correlation with the total thyroidectomy for toxic goiter which is the most frequent operation associated with TELN and PELN injury, with a Pearson correlation 0.76 and 0.73, and a p value of 0.004 and 0.007 respectively.

**Table 2:** Nerve injury risk whether nerve identified or not TRLNP: Transient Recurrent Laryngeal Nerve PRLNP: Permanent Recurrent Laryngeal Nerve TELNP: Transient External Laryngeal Nerve PELNP: Permanent External Laryngeal Nerve

Groups	PRLNP (n)	%	TRLNP (n)	%	PELNP (n)	%	TELNP (n)	%	Total (n)	%
G1	0	0	5	6.6%	1	1.3%	8	10.6%	14	18.6%
G2	0	0	3	4%	2	2.6%	3	4%	8	10.6%
Total	0		8		3		11		22	14.6%

**Table 3:** Incidence of nerve injury in relation to type of surgery in G1 patients TRLNP: Transient Recurrent Laryngeal Nerve PRLNP: Permanent Recurrent Laryngeal Nerve TELNP: Transient External Laryngeal Nerve PELNP: Permanent External Laryngeal Nerve

Variables	Total thyroidectomy				Hemi thyroidectomy				Lobectomy				Total	
	Toxic (n)	%	Non toxic (n)	%	Toxic (n)	%	Non toxic (n)	%	Toxic (n)	%	Non toxic (n)	%	(n)	%
T RLN	3	4%	0	0	1	1.3%	0	0	0	0	1	1.3%	5	6.6%
P RLN	0	0	0	0	0	0	0	0	0	0	0	0	0	0
T ELN	5	6.6%	2	2.6%	1	1.3%	0	0	0	0	0	0	8	10.6%
P ELN	1	1.3%	0	0	0	0	0	0	0	0	0	0	1	1.3%
Total	9	11.9 %	2		2		0	0	0	0	1		14	18.6%

**Table 4:** Incidence of nerve injury in relation to type of surgery in G2 patients TRLNP: Transient Recurrent Laryngeal Nerve PRLNP: Permanent Recurrent Laryngeal Nerve TELNP: Transient External Laryngeal Nerve PELNP: Permanent External Laryngeal Nerve

Variables	Total thyroidectomy				Hemi thyroidectomy				Lobectomy				Total	
	Toxic (n)	%	Non toxic (n)	%	Toxic (n)	%	Non toxic (n)	%	Toxic (n)	%	Non toxic (n)	%	(n)	%
T RLN	0	0	2	2.6%	0	0	0	0	0	0	1	1.3%	3	4%
P RLN	0	0	0		0	0	0	0	0	0	0	0	0	0
T ELN	2	2.6%	1	1.3%	0	0	0	0	0	0	0	0	3	4%
P ELN	1		1	1.3%	0	0	0	0	0	0	0	0	2	2.6%
Total	3		4		0		0		0		1	1.3%	8	10.6

**Table 5:** Chi-Square tests results show association between the nerve injury rate in relation to the nerves had been identified or not df: Degree of freedom

Data	Value	df	P-value
Pearson chi-square	1.588	2	0.452
Likelihood ratio	1.546	2	0.462
Linear-by-linear association	0.264	1	0.607
Number of valid cases	22		

## Discussion

Theodor Kocher was responsible for reducing the thyroidectomy's surgical mortality from 14.8% in 1882 to a final level of less than 0.18% in 1898. His exacting methods led to a rate of recurrent nerve damage comparable to that of modern surgeons [10]. Later, in 1938, Lahey examined practically every aspect of the RLN. He claimed that careful dissection reduced the amount of RLN injuries [11]. Injury to the recurrent laryngeal nerve(s) can occur anywhere along its course in the vicinity of the thyroid gland. It is usually accidentally ligated and divided in an attempt to control excessive bleeding in the RLN vicinity or it may excessively stretch during surgery for a large thyroid gland [12]. This study showed that G2 with meticulous dissection away from the site of nerve course has an overall risk of nerve injury lower than G1, although the result is statistically insignificant and no valuable association between nerve identification and the rate of nerve injury was found. Both groups show no permanent RLNP, which means that it’s possible not to search for the recurrent laryngeal nerve during thyroidectomy if we dissect it very close to the thyroid gland capsule. This result agreed with the study's results regarding the same policy of non-exploration for RLN [13-29]. While other authors suggest routine exploration for the RLN to avoid or reduce the injury [14]. It is possible to identify this nerve with a high rate of accuracy by comprehending the three structural variations of the distal segment of the external branch of the superior laryngeal nerve (EBSLN) and its relationship to the inferior constrictor muscle. During thyroid surgery, the EBSLN should be examined, and identification is usually possible. The larynx function is kept at its peak by maintaining the EBSLN [16]. 

Incidence of external laryngeal nerve palsy is higher in G1 than in G2; this might be due to over-dissection to find the nerve with variable anatomical location and vulnerability of the nerve. Because the EBSLN is present between the inferior pharyngeal muscle fibers in around 20% of instances, several writers stressed that performing a muscle dissection could increase the chance of nerve injury. As a result, they advised against doing so [17]. The superior pole vessels should not be ligated in bulk, but rather individually divided, low on the thyroid gland, and dissected laterally to the cricothyroid muscle [4]. Even for skilled surgeons, it can be challenging to visually recognize and separate external superior laryngeal nerve (ESLN) from fibrillar structures that resemble nerves [18]. It is safe and may prevent ESLN harm to individually ligate superior thyroid arteries near the thyroid capsule without first identifying the ESLN [23,28]. We believe that the type of surgery performed in patients with toxic goiters in both groups carries a higher risk of nerve injury compared to other types of surgeries. This finding is consistent with the findings of Zambudio et al. [23] as they think that hyperthyroidism and goiter size are the two primary independent risk factors for the development of complications. Hyperthyroidism is linked to a 2.5-fold higher risk of RLNP than in thyroid surgery with euthyroidism. Liu et al. [24] disagreed with this theory, however, they underlined that the type of thyroidectomy did not appear to have an impact on permanent recurrent laryngeal nerve palsy. We think that the surgeon's experience has a lot of advantages in doing safe and very low incidence of nerve injury. Low-volume surgeons (those who do less than 30 cases per year) are more likely to experience persistent nerve damage [19-21,29]. Duclos and colleagues [21] advocate arguing against the idea that achieving or maintaining one's optimal performance in thyroid surgery may be done merely by gaining experience. There should be more research done on the factors causing very experienced surgeons to perform poorly.

When a supervised trainee conducts the procedure, there is no change in the incidence of RLNP compared to prior trials [26,27]. Although we didn’t use nerve stimulator in our center because its already not available in our country, we believe that its use did not affect the outcome regarding injury risk to the nerves. This idea is agreed on by Loch-Wilkinson et al. [29] who asserted that the experienced surgeon does not clearly benefit from nerve stimulation for intermittent nerve monitoring during total thyroidectomy in terms of nerve identification, functional testing, or damage prevention. The RLN damage incidence was less than 1% when performed by skilled thyroid surgeons, however the IONM did not significantly improve postoperative results [30].

The study has some limitations such as small size and only one surgical intervention being evaluated. Further studies with a larger sample size should be carried out.

## Conclusions

External laryngeal nerve injury during thyroid surgery is unavoidable at times regardless of the procedure used, particularly total thyroidectomy for toxic goiters. However, with an expert surgeon in thyroid surgery and careful dissection close to the thyroid capsule even without definite visualization of the nerve, it may be possible to reduce the risk of recurrent laryngeal nerve injury.
